# Safe Sleep Practices of Kansas Birthing Hospitals

**Published:** 2018-02-28

**Authors:** Carolyn R. Ahlers-Schmidt, Christy Schunn, Cherie Sage, Matthew Engel, Mary Benton

**Affiliations:** 1 Univeristy of Kansas School of Medicine-Wichita, Wichita, KS; 2 Kansas Infant Death and SIDS Network, Wichita, KS; 3 Safe Kids Kansas, Topeka, Kansas

**Keywords:** infant mortality, organizational policy, hospital, pediatrics, guidelines

## Abstract

**Background:**

Sleep-related death is tied with congenital anomalies as the leading cause of infant mortality in Kansas, and external risk factors are present in 83% of these deaths. Hospitals can impact caregiver intentions to follow risk-reduction strategies. This project assessed the current practices and policies of Kansas hospitals with regard to safe sleep.

**Methods:**

A cross-sectional survey of existing safe sleep practices and policies in Kansas hospitals was performed. Hospitals were categorized based on reported delivery volume and data were compared across hospital sizes.

**Results:**

Thirty-one of 73 (42%) contacted hospitals responded. Individual survey respondents represented various hospital departments including newborn/well-baby (68%), neonatal intensive care unit (3%) and other non-nursery departments or administration (29%). Fifty-eight percent of respondents reported staff were trained on infant safe sleep; 44% of these held trainings annually. High volume hospitals tended to have more annual training than low or mid volume birth hospitals. Thirty-nine percent reported a safe sleep policy, though most of these (67%) reported never auditing compliance. The top barrier to safe sleep education, regardless of delivery volume, was conflicting patient and family member beliefs.

**Conclusions:**

Hospital promotion of infant safe sleep is being conducted in Kansas to varying degrees. High and mid volume birth hospitals may need to work more on formal auditing of safe sleep practices, while low volume hospitals may need more staff training. Low volume hospitals also may benefit from access to additional caregiver education materials. Finally, it is important to note hospitals should not be solely responsible for safe sleep education.

## Introduction

In 2016, the state of Kansas had an infant mortality rate (IMR) of 5.9 per 1,000 live births.^1^ While infant mortality in Kansas has reached the Healthy People 2020 target of 6.0, sudden unexplained infant deaths (SUID) are tied with congenital anomalies for the leading cause of death.^1^,^2^ SUID includes deaths from unknown cause and sleep-related deaths, such as Sudden Infant Death Syndrome (SIDS) and accidental suffocation or strangulation in bed.^1^ In 2014, 83% of deaths attributed to SIDS had one or more factors contributing to an unsafe sleep environment.^3^

The American Academy of Pediatrics (AAP) recommendations for a safe infant sleeping environment delineate a number of modifiable factors to reduce the risk of sleep-related infant deaths.^4^ Factors include back sleep only, room-sharing without bed-sharing, use of a firm sleep surface, keeping soft bedding and other items out of the crib, and avoiding infant overheating. The AAP also suggested health care providers, especially those in hospitals, model safe sleep recommendations.

In concert with AAP efforts, the Collaborative Improvement and Innovation Network to Reduce Infant Mortality (IM CoIIN) began a multiyear national movement in 2014 to reduce infant mortality and improve birth outcomes.^5^ One of the six strategic areas of focus includes improving safe sleep practices. The Kansas Infant Death and SIDS Network (www.kidsks.org), in collaboration with Safe Kids Kansas (www.safekidskansas.org), is leading the Kansas Safe Sleep CoIIN Group’s multi-pronged effort to increase safe sleep practices. Efforts include building on previous work^6^–^8^ with maternal and infant health providers to enhance delivery of anticipatory guidance related to safe sleep.

Postpartum education on infant sleep can be provided in hospital settings through direct education and modeling proper infant safe sleep strategies;^9^–^11^ both impact adherence to safe sleep recommendations at home.^9^,^12^ For some groups at high risk for sleep-related death, such as African Americans,^13^ hospitals may be the main or only source of safe sleep education.^14^,^15^ However, hospitals are not consistent in their practice of the AAP infant safe sleep recommendations.^16^

Hospital healthcare providers involved in infant care have a unique opportunity to influence infant safe sleep. The purpose of this project was to assess delivering hospitals’ activities related to safe sleep, specifically with regard to policies and practice.

## Methods

This was a cross-sectional survey of Kansas hospitals that were identified as having at least one delivery in the year prior to the study (2015). The Safe Sleep CoIIN group partnered with the Kansas Hospital Association (KHA), which sent the survey to these hospitals across the state.

No validated survey was identified; therefore, an instrument was created to ascertain current hospital practices related to infant safe sleep. Thirteen questions were developed by the CoIIN group. The final survey was reviewed for readability and content validity by the Medical Society of Sedgwick County Safe Sleep Taskforce, a group consisting of physicians, community members, and researchers. Surveys asked about safe sleep training for hospital staff, safe sleep hospital policy, following of AAP recommendations in the hospital, and education provided to patients. For those hospitals that endorsed having a safe sleep policy, respondents were asked about the contents of that policy. Finally, participants were asked about barriers to improving safe sleep practices in their hospitals and additional comments were invited at the end of the survey.

A cover letter was developed requesting the anonymous survey be forwarded to the most appropriate person, as only one response was allowed per hospital. In January 2016, KHA emailed the cover letter and a SurveyMonkey_®_ link to 74 hospitals. One reminder was sent in the same manner.

Responses were summarized with frequencies and percentages reported. For analysis, hospitals were divided into empirical categories based on reported volume. Differences between results for volume groups were evaluated using chi-squared tests. Number of items provided to parents to support safe sleep were compared across volume groups using the Kruskal-Wallis H-test. Analysis was performed using SPSS 23 [IBM Corp, Armonk, NY].

## Results

Thirty-one hospitals provided responses (42% response rate). Different hospital departments responded to the survey with most (68%) coming from the newborn/well-baby unit, and others coming from the neonatal intensive care unit (NICU; 3%) and other non-nursery departments (29%), including general staff and administrators from small rural hospitals. The median number of reported deliveries for surveyed hospitals was 100, but ranged from as high as 1800 to as low as zero. One respondent commented they “do not…deliver babies unless they drop on our door step”. Fourteen hospitals (45%) delivered between 0 and 49 infants annually (low volume), 9 (29%) delivered between 50 and 500 (mid volume), and 8 (26%) delivered more than 500 (high volume).

In regard to training, 18 responding hospitals (58%) reported they provided infant safe sleep training to staff. Of these, 8 (44%) reported annual trainings. High volume hospitals (63%) were more likely to hold annual training as compared to low (7%) or mid (22%) volume hospitals (χ^2^ (4, N =31) = 13.3; p = 0.01).

Thirty-nine percent of hospitals (n = 12) reported a safe sleep policy. Of those, 10 (83%) had revised their policy following the 2011 guideline update. Mid (80%) and high volume hospitals (100%) reported keeping their policies up-to-date more frequently than low volume hospitals (50%), though this difference was not statistically significant (χ^2^ (2, N =12) = 2.6; p = 0.267). All hospitals with a policy reported that keeping soft items out of cribs was addressed and the majority addressed avoiding co-sleeping (Table 1). Policies differed by hospital size, with low volume hospitals less likely to encourage rooming in or back sleeping (χ^2^ (2, N = 12) = 8.8; p = 0.012). Avoiding co-sleeping of multiple birth siblings (i.e., twins) was lacking from 50% of policies.

Across all hospitals, discharge instructions (n = 23, 74%) and printed materials (n = 21, 68%) were the most common resources provided to patients (Table 2). Low volume hospitals had fewer resource provisions overall (median = 1.5) as compared to mid (median = 4) and high volume (median = 5) (H(2) = 9.5, p = 0.009); no low volume hospitals reported offering a safe sleep instructional video viewing and only 4 (29%) reported offering educational classes on newborn care. Notably, more mid volume hospitals (67%) offered viewing of a safe sleep video, than high volume hospitals (38%; χ^2^ (2, N = 31) = 12.2; p = 0.002).

Most hospitals (71%) reported sometimes or always asking parents if they had a safe crib at home. Few (23%) reported referring parents to crib distribution programs. Two-thirds of hospitals (67%) reported never auditing compliance with their infant safe sleep policy. Two (13%) reported auditing weekly and two (13%) reported auditing annually. The remaining site (7%) reported occasional “spot checks”.

Respondents reported how frequently safe sleep recommendations were followed by staff (Table 3). Most hospitals reported always utilizing tight fitting sheets (96%) and back positioning (78%). Only one hospital (7%) reported “never” putting infants in a crib. Low volume hospitals used wearable blankets infrequently (50% reported never using), though most high volume hospitals (88%) used wearable blankets at least sometimes. A majority of hospitals (63%), irrespective of volume, reported always keeping toys and diapers out of cribs. Fewer (22%) reported always keeping blankets out of cribs.

A number of barriers to improving infant safe sleep were reported (Table 4). The top barrier, irrespective of hospital volume, was conflicting patient and family member beliefs. Lower volume hospitals also reported lack of appropriate educational materials, low awareness of infant safe sleep practices among nursing or medical staff, and language barriers as impediments to improving infant safe sleep practices. Mid volume hospitals reported language barriers, competing demands for staff, and nursing staff not always following infant safe sleep practices. High volume hospitals reported language barriers and nursing staff not always following safe sleep practices as their other major impediments.

## Discussion

As evident by the survey responses, hospital promotion of infant safe sleep is being conducted in Kansas to varying degrees. While the sample size is small, a number of interesting findings emerge. More than half of responding hospitals reported offering safe sleep training for staff, with high volume hospitals more likely to conduct annual trainings than mid or low volume hospitals. In contrast, fewer hospitals reported a safe sleep policy. All reported policies address keeping soft items out of cribs and high volume hospitals’ policies were more complete in terms of addressing the AAP guidelines. However, most hospitals reported never auditing policy compliance. Across all hospitals, discharge instructions and printed materials were the most common resources provided to parents. However, the quality and content of the discharge instructions were not assessed. The new AAP Safe Sleep guidelines^4^ and the National Action Partnership to Promote Safe Sleep (NAPPSS; www.nappss.org) are advocating for enhanced conversations with parents regarding infant sleep to better address individual barriers. Finally, while most hospitals reported usually asking parents if they had a safety-approved crib available, very few refer parents to crib distribution programs.

Barriers to providing safe sleep education differed somewhat by hospital volume. However, regardless of volume, parent and family member’s beliefs are perceived to be a major barrier to improving infant safe sleep practices across hospitals. There are cultural norms to overcome with regard to infant safe sleep. Further, language barriers often were cited as a barrier by mid and high volume hospitals, while not endorsed as frequently in low volume hospitals. Mid and high volume hospitals also reported nursing staff not always following safe sleep practices. However, unlike low volume hospitals, no mid or high volume hospitals reported a lack of awareness of infant safe sleep practices.

There may be different remedies for improving infant safe sleep practice depending on the hospital birth volume. In reviewing the barriers to safe sleep implementation, low volume hospitals appeared to need more training and educational materials, while mid and high volume hospitals may need to work more on implementation of safe sleep practices. This is concordant with studies aimed at improving infant sleep environments in a hospital setting. One study in a large hospital (more than 6,000 annual deliveries) observed whether infants met the AAP guidelines for safe sleep.^6^ Prior to implementing a bundled intervention, only 25% of infants were observed in a safe sleep environment. Another study reported that while 97% of nurses knew the AAP recommendations for safe sleep, only 67% agreed, and only 29% of the infants were found lying in the supine position, compared with 55% side-lying and 16% in the prone position.^17^

Programs that incentivize hospitals or offer providers continuing education credits may increase the likelihood that safe sleep initiatives are introduced. For example, Cribs for Kids, a national safe sleep initiative, established a certification program to encourage hospitals to promote safe infant sleep.^18^ Three levels of certification are available: gold, silver, and bronze. To date, only two Kansas hospitals have received this certification, yet many of the survey respondents indicated activities that would qualify them for at least bronze certification. The Kansas Safe Sleep CoIIN group should explore ways to ensure hospitals are aware of the program and support efforts to obtain or renew certification.

While the results of this study may not be representative of all birthing hospitals in Kansas, the response rate of 42% is indicative of a good cross-section of hospitals, especially from respondents of newborn/well-baby departments. Additionally, while it may be desirable to have more respondents from mid and high volume hospitals, the inclusion of a sizable percentage of low volume hospitals provided unique insight into infant safe sleep practices of small and rural hospitals. This survey provides a solid first step to develop interventions and/or tools for the promotion of infant safe sleep.

Safe sleep education and promotion occur at varying levels in Kansas hospitals and interventions to improve safe sleep promotion appears to be associated with birthing volume. High and mid volume hospitals should adopt more formal auditing of safe sleep practices, implement tools to work across language barriers, and support nurse promotion of AAP guidelines. Low volume hospitals may benefit from staff training and access to additional caregiver education materials, such as showing a safe sleep video prior to discharge. Finally, hospitals should not be solely responsible for safe sleep education. Pre- and postnatal care providers and community programs also should promote consistent infant safe sleep messages to enhance the likelihood families will follow risk reduction recommendations.

## Figures and Tables

**Table 1:** Policies and resources devoted to safe sleep for hospitals with a policy (n = 12), n (%).

Which items are explicitly included in your department’s safe sleep policy/guideline?	Birth volume*		
	Low (n=2)	Mid (n=5)	High (n=5)
Avoid co-sleeping	2 (100)	4 (80)	5 (100)
Avoid co-sleeping of multiple-birth siblings	1 (50)	1 (20)	4 (80)
Avoid infant overheating	2 (100)	3 (60)	5 (100)
Back sleep only for infants	1 (50)	4 (80)	5 (100)
Encourage rooming in	1 (50)	4 (80)	5 (100)
Keep soft items out of crib	2 (100)	5 (100)	5 (100)

* Low: <50 births, Mid: 50–500 births, High: >500 births

**Table 2:** Educational/information items devoted to safe sleep by number of births, n (%).

Which of the following education, information or items devoted solely or primarily to safe sleep practices does your department provide?	Birth volume*		
	Low (n=14)	Mid (n=9)	High (n=8)
Brochures and other printed materials	7 (50)	6 (67)	8 (100)
Discharge instructions on safe sleep	8 (57)	8 (89)	7 (88)
Educational messages on products (t-shirts, mugs)	2 (14)	1 (11)	1 (13)
In-house wearable blanket	3 (21)	3 (33)	5 (63)
Newborn classes	4 (29)	6 (67)	5 (63)
Offered viewing of safe sleep instructional video	0 (0)	6 (67)	3 (38)
Posters	2 (14)	3 (33)	3 (38)
Required viewing of safe sleep instructional video	1 (7)	2 (22)	0 (0)
Take home wearable blanket	1 (7)	1 (11)	4 (50)

* Low: <50 births, Mid: 50–500 births, High: >500 births

**Table 3:** Frequency of staff following safe sleep recommendations for hospitals with any births (n=27), n (%).

Safe Sleep Recommendation	Birth volume*								
	Low (n=10)			Mid (n=9)			High (n=8)		

	Always	Sometimes	Never	Always	Sometimes	Never	Always	Sometimes	Never
In a crib	7 (70)	2 (20)	1 (10)	9 (100)	0 (0)	0 (0)	7 (88)	1 (13)	0 (0)
On the back/supine position	8 (80)	1 (10)	1 (10)	7 (78)	2 (22)	0 (0)	6 (75)	2 (25)	0 (0)
Tight fitting sheet	9 (100)	0 (0)	0 (0)	8 (89)	0 (0)	1 (11)	8 (100)	0 (0)	0 (0)
No blankets	1 (10)	7 (70)	2 (20)	2 (22)	6 (67)	1 (11)	3 (38)	5 (63)	0 (0)
No toys or extra diapers in the crib	7 (78)	2 (22)	0 (0)	5 (56)	4 (44)	0 (0)	5 (63)	3 (38)	0 (0)
Sleepsack/wearable blanket	2 (20)	3 (30)	5 (50)	2 (22)	4 (44)	3 (33)	4 (50)	3 (38)	1 (13)

Note: Responses of “Not Sure” were treated as missing data; percent is calculated as percent of similar sized hospitals.

* Low: <50 births, Mid: 50–500 births, High: >500 births

**Table 4:** Barriers to improving safe sleep, n (%)

What do you think are strong barriers to improving safe sleep practices in your Department?	Birth volume*		
	Low (n=14)	Mid (n=9)	High (n=8)
Patient/family beliefs	5 (36)	6 (67)	5 (63)
Language barriers	2 (14)	5 (56)	3 (38)
Nursing staff don’t always follow guidelines	1 (7)	3 (33)	3 (38)
Competing staff priorities	2 (14)	4 (44)	1 (13)
Lack of appropriate educational materials	3 (21)	2 (22)	1 (13)
Low awareness of safe sleep practices	3 (21)	0 (0)	0 (0)
Staff belief that co-sleeping improves bonding	1 (7)	1 (11)	1 (13)
Physicians don’t always follow guidelines	0 (0)	2 (22)	1 (13)
Not enough time to educate patients during stay	1 (7)	1 (11)	0 (0)

* Low: <50 births, Mid: 50–500 births, High: >500 births
